# Maternal dietary taurine supplementation improves intestinal health of lambs via modulating gut microbiota and barrier function

**DOI:** 10.3389/fmicb.2026.1662296

**Published:** 2026-02-16

**Authors:** Guoqiang Huo, Xuan Liu, Jianrong Huo, Jinxin Feng, Xiaoyi Zhao, Yating Li, Bo Wang, Junxing Zhao

**Affiliations:** 1 College of Animal Science, Shanxi Agricultural University, Jinzhong, Shanxi, China; 2 Shanxi Key Laboratory of Animal Genetics Resource Utilization and Breeding, Jinzhong, Shanxi, China; 3 State Key Laboratory of Animal Nutrition and Feeding, Department of Animal Nutrition and Feed Science, College of Animal Science and Technology, China Agricultural University, Beijing, China; 4 Yazhouwan National Laboratory, Sanya, China

**Keywords:** diarrhea, gut microbiota, intestinal barrier function, maternal supplementation, neonatal lambs, taurine

## Abstract

Pre-weaning lambs are often at risk of diarrhea, and thus, intestinal development and normal function are closely related to their health and survival. This study investigated whether maternal dietary supplementation of taurine (TAU) during gestation is associated with distinct microbial community features and alterations in microbial functional potential in the offspring’s gut microbiota, and whether these microbiota-associated alterations were accompanied by improvements in intestinal development, barrier function, immune homeostasis, and antioxidant capacity. Ewes were fed with different concentrations of taurine (0, 0.1 and 0.2%) during gestation, and lambs’ gut microbiota composition and intestinal barrier function were determined. The results showed that lambs’ body weights at day 15 after birth were elevated by maternal dietary taurine intake. Moreover, maternal taurine supplementation was associated with shifts in beneficial bacterial groups, including members of *Lachnospiraceae* (e.g., *Coprococcus*, Ruminococcus_gauvreauii_group, Lachnospiraceae_FE2018_group, *Blautia*), as well as *Ruminococcus* and *Eubacterium*, together with alterations in microbial functional potential. These microbiota-associated alterations were accompanied by increased villus height, reduced crypt depth, and upregulation of ZO-1, Claudin-1, Occludin and Mucin2 (MUC2) expression, along with reduced Notch2 expression. Importantly, dietary taurine supplementation downregulated IL-1β, IL-6, NF-κB and malondialdehyde (MDA) levels, and upregulated IL-10, Superoxide dismutase (SOD) and glutathione (GSH) contents. In conclusion, maternal dietary taurine supplementation during gestation was associated with distinct microbiota-related features and improved intestinal barrier integrity in pre-weaning lambs, which may contribute to increased body weight. Despite the relatively limited sample size, the observed associations were directionally consistent across microbial and host-level analyses; future studies with larger cohorts will be necessary to confirm and extend these findings.

## 1 Introduction

Neonatal diarrhea represents a major health challenge that compromises early survival and growth performance of lambs. It is commonly associated with gut barrier dysfunction, impaired nutrient absorption, and weakened immune responses, leading to reduced average daily gain, suboptimal weaning weights, and ultimately compromised disease resistance (Aghakeshmiri et al., 2017; Jessop et al., 2024). Studies have shown that diarrhea is not only caused by pathogenic infections (*Escherichia coli* and *Clostridium perfringens*), but also closely linked to immature intestinal development, microbial dysbiosis, and insufficient colonization of beneficial microbes (Liu et al., 2022; Du et al., 2023). The intestinal microbiota contributes to establishing immune homeostasis and metabolic function in neonates, and its composition and stability are critical determinants of intestinal health and host resilience (Malmuthuge et al., 2015). Thus, modulating gut microbiota, particularly through enhancing the abundance of beneficial bacteria, has been employed to alleviate diarrhea and potentiate growth performance (Bi et al., 2019; Quijada et al., 2020). Neonate’s body weight (BW) is a key indicator of growth and development potential, reflecting nutrient utilization efficiency and intestinal functional status. Şahin (2022) reported that lambs with higher birth and weaning weights exhibited improved post-weaning growth performance. Similarly, Mirzaei et al. (2020) found that appropriate weaning strategies enhanced growth performance and metabolic outcomes in calves.

As a prevalent free amino acid in mammals, taurine possesses various physiological functions, including antioxidative and anti-inflammatory actions, osmotic balance, and membrane stabilization (Jong et al., 2012; Ma et al., 2022; Zhao et al., 2023). In animal husbandry, taurine has been used as conditionally essential supplement. For example, dietary taurine supplementation in broiler enhances intestinal barrier function, reduces inflammation, improves antioxidant capacity, and ultimately promotes growth and disease resistance (Han et al., 2020). Similarly, taurine supplementation in weaned piglets is shown to improve intestinal tissue development and barrier protection, enhance antioxidant capacity, and promote growth performance (Tang et al., 2018). Under heat stress conditions, combined supplementation of taurine and folic acid in lambs enhance antioxidant and immune functions, modulate rumen microbial composition, and reduce the harmful effects of heat stress (Li et al., 2024). Considering the immature digestive function of neonates, direct feeding of taurine may result in low absorption efficiency. Maternal nutritional status profoundly affects early development of the fetus and neonates by influencing metabolic pathways, immune programming, and gut microbiota colonization (Grant et al., 2023), and thus, offers an effective strategy to deliver functional nutrients to the offspring. In a streptozotocin-induced diabetic rat model, maternal taurine supplementation has demonstrated protective effects of oxidative stress caused by diabetes in both dams and their embryos (Shivananjappa, 2012). In pigs, maternal taurine supplementation improves offspring physiological status (Xu et al., 2019). Whether maternal taurine supplementation influences neonatal lambs’ gut microbiota composition and health remains undefined.

The connection between maternal diet, gut microbial community, and host immunity is increasingly recognized as a key mechanism shaping offspring’s intestinal development and barrier function. Given taurine’s regulatory function on the barrier and microbiota in lambs (Li et al., 2024), its potential impact on the neonatal lambs’ gut health under maternal intervention warrants further investigation. Our study evaluated the effects of maternal dietary taurine intake on the intestinal microbiota composition and function, barrier function, and immune status of neonatal lambs, and explored the potential mechanism.

## 2 Material and method

### 2.1 Animals and experimental design

This research was performed under the approval of the Institutional Animal Care and Use Committee of Shanxi Agricultural University (sxnd202028). A total of 18 Hu ewes (18 months, 50 kg ± 1 kg) were randomly selected, equally assigned into 3 groups (*n* = 6) and fed corresponding diet containing different concentration of taurine (purity ≥ 99%, Shanghai Jin Sui Biotechnology Co., Ltd.): control group (0% taurine), 0.1% TAU group (0.1% taurine) and 0.2% TAU group (0.2% taurine). The basal diets were formulated to be isonitrogenous and isoenergetic, taurine was administered orally by gavage, and ewes had unrestricted access to both feed and water. Estrus synchronization was performed to ensure all ewes were inseminated simultaneously using semen from Dorper rams.

Feed nutritional content was evaluated following standard procedures (AOAC, 2023), and the basal diet’s complete nutrient profile is presented in Table 1. Samples were freeze-dried for 72 h and ground to pass a 1 μm sieve. Analyses included dry matter (DM) by drying at 105 °C for 2 h (method 930.15); crude protein (CP) via the Kjeldahl method (method 980.10), calculated as N × 6.25; ether extract (EE) by Soxhlet extraction (method 920.39); and ash by combustion at 550 °C for 2 h (method 942.05). Neutral detergent fiber (NDF) and acid detergent fiber (ADF) were determined following Van Soest et al. (1991) using heat-stable *α*-amylase and sodium sulfite.

**Table 1:** Feed ingredients and nutrient levels of pregnant ewes (DM basis, %).

Items	Pregnant ewes
Ingredients	
Corn	18.00
Soybean meal	9.33
Wheat bran	3.83
Premix	1.67^a^
Baking soda (NaHCO_3_)	0.33
Salt (NaCl)	0.17
Grass hay	66.67
Total	100.00
Nutrient levels	
DM	88.63
CP	12.68
EE	1.77
OM	79.88
NDF	36.64
ADF	26.11

DM = dry matter; CP = crude protein; EE = ether extract; OM = organic matter; NDF = neutral detergent fiber; ADF = acid detergent fiber.

a One kilogram of pregnant ewe premix contains the following substances: 260000 IU Vitamin A, 100000 IU Vitamin D3, 1800 IU Vitamin E, 200 g Ca, 45 g Mg, 30 g P, 12 g K, 5 g Na, 2.4 g Zn, 2.2 g Mn, 350 mg Cu, 60 mg I, 27 mg Co, 14 mg Se.

### 2.2 Sample collection

The following sampling and analyses were conducted with six biological replicates per group, including ewe and lamb serum taurine concentration, lamb body weight, lamb intestinal gene expression (qPCR), and lamb serum immune and antioxidant parameters. Blood was sampled from the ewe’s jugular vein during early, mid, and late gestation, as well as from offspring for serum collection. Aliquots of serum were prepared and frozen at −80 °C for future analyses. To minimize potential sex-related variability, only male lambs were selected for downstream analyses. Hu sheep is a prolific breed with a high incidence of twin and multiple births, which ensured the availability of sufficient male lambs per treatment group without introducing selection bias. Male lambs were weighed, euthanized, and sampled at 15 days after birth. Prior to euthanasia, lambs were deeply anesthetized by intravenous injection of sodium pentobarbital (30 mg/kg body weight) via the jugular vein to ensure complete loss of consciousness and absence of pain. Once deep anesthesia was confirmed by the absence of corneal reflex and response to toe pinch, euthanasia was performed by exsanguination. After euthanasia, intestinal tissues were collected from multiple segments for histological evaluation, including the duodenum, jejunum, ileum and colon. For microbiome profiling and gene expression analyses, samples were intentionally collected from the jejunum only. This segment was selected because it represents a functionally central region for nutrient absorption and early-life intestinal barrier and immune regulation, and is directly involved in taurine uptake via specific intestinal transport systems, while maintaining analytical focus and statistical robustness (Anderson et al., 2009). Jejunum segments (approximately 3–4 mm) were fixed in 4% paraformaldehyde solution (Biosharp, BL539A, Hefei, China) for paraffin embedding and histological analysis. Additional jejunal tissues and digesta were immediately stored at −80 °C for subsequent molecular analyses.

### 2.3 Hematoxylin and eosin (H&E) staining

For histomorphometric analysis, jejunal tissues were collected from six lambs per group. After fixation and paraffin embedding, 2–3 tissue sections per animal were prepared and stained with hematoxylin and eosin (HE). Within each section, at least 10 complete, well-oriented villus-crypt units were randomly selected and measured. The villus height and crypt depth for each animal were calculated as the mean of all measured units. Dehydration of the fixed tissues was performed through increasing concentrations of ethanol (75, 85, 95, and 100%) with 2 h at each step, cleared sequentially in ½ ethanol:½ xylene, xylene I, and xylene II (30 min each), and then infiltrated with ½ xylene:½ paraffin, low-melting paraffin (58 °C), and high-melting paraffin (60 °C) for 3 h each. After embedding, the tissues were sliced to a thickness of 5 μm by means of a microtome (RM2265, Leica, Wetzlar, Germany) and mounted on glass slides. Prior to staining, the sections were dewaxed in xylene I and II (10 min each), rehydrated through a descending ethanol series (100, 95, 85, 70, and 50%, 2 min each), and stained with HE by the instructions (Solarbio, G1120, Beijing, China). After staining, the sections underwent dehydration, xylene clearing, and neutral balsam mounting. Images were captured under microscope (DMi8, Leica, Wetzlar, Germany).

### 2.4 Alcian blue-periodic acid-schiff (AB-PAS) staining

5-μm-thick tissue sections were dewaxed in xylene I and II (10 min each), followed by rehydration through a stepwise ethanol series (100, 95, 85, 70, and 50%), with 2 min at each concentration. For each animal, 2–3 tissue sections were analyzed, and within each section, at least 10 representative microscopic fields were randomly selected for evaluation of goblet cells. After rehydration, according to the instructions (Solarbio, G1285, Beijing, China), AB-PAS staining was conducted according to standard procedures. Tissue sections were first immersed in Alcian blue solution for 15 min, distilled water was used to rinse the samples. They were then treated with 0.5% periodic acid for 10 min, rinsed again, and subsequently exposed to Schiff reagent for 15–20 min. After coloration, the sections were washed in running water for 5–10 min. After section mounting and drying, images were captured microscopically (DMi8, Leica, Wetzlar, Germany).

### 2.5 Enzyme-linked immunosorbent assay (ELISA)

A sheep-specific ELISA kit (MEIMIAN, MM-8115401, Jiangsu, China) was applied to measure taurine levels in serum. In addition, the concentration of bile acids (Nanjing Jiancheng, E003-2-1, Nanjing, China) in the serum was determined. The jejunal tissue was weighed and ground into powder. Then, saline was added at 1:9 (w/v), and commercial assay kits (Nanjing Jiancheng, Nanjing, China) were used to determine the content of glutathione (GSH, A006-2-1), superoxide dismutase (SOD, A001-3-2), and malondialdehyde (MDA, A003-1-2) in the samples.

### 2.6 Quantitative real-time PCR (qPCR) analysis

TRIzol reagent (Invitrogen, 15596026CN, US) was used to extract total RNA from jejunal tissue as per the supplier’s instructions. After that, a reverse transcription kit (Takara, RR037A) was employed to synthesize cDNA from RNA, qRT-PCR was conducted using qPCR reagents (Mei5bio, MF013-01, Beijing, China). Relative mRNA expression was calculated using the 2^–ΔΔCt^ method, with *β*-actin as the reference gene. Briefly, the threshold cycle (Ct) values of target genes were first normalized to the Ct of β-actin to obtain ΔCt values. The ΔCt of each sample was then compared to that of the control group to calculate ΔΔCt, and relative gene expression was determined as 2^−ΔΔCt^, representing the fold change relative to the control (Livak and Schmittgen, 2001). Primer information is detailed in Table 2. All primers were designed based on gene sequences available in the NCBI database and synthesized by Sangon Biotech Co., Ltd. (Shanghai, China).

**Table 2:** Primer sequences used for qRT-PCR analysis.

Gene	Direction	Sequence (5′-3′)
*Muc2*	Forward	TCACCTGCCCTGACTTTGAC
	Reverse	TGCGAAATCTCCCTCGTGAC
*Notch2*	Forward	GGACTGCCTTGATGTGCCA
	Reverse	CACCATTCTGGCAACGGTTC
*ZO-1*	Forward	AATACATTGAGGTCACCGAGTA
	Reverse	GATTAGGCAAGGAAAGGCAC
*Claudin-1*	Forward	AGAAGATAGCCCTGCAGCCAA
	Reverse	CCTCTCCTTTGTTAAAACTAAGTC
*Occludin*	Forward	AGTGGTAACTTGGAGACGCTTTC
	Reverse	CCTCCCGTCGTGTAGTCTGTT
*IL-6*	Forward	TGCAGTCCTCAAACGAGTGG
	Reverse	TACCACAATCATGGGAGCCG
*IL-1β*	Forward	AGTGCCTATCCTCGGACCC
	Reverse	GGCGCACTAACCCAAAGGA
*β-actin*	Forward	CTTCCAGCCTTCCTTCCTGG
	Reverse	GCCAGGGCAGTGATCTCTTT

### 2.7 Western blot analysis

Jejunal total protein was isolated using RIPA buffer (Bosterbio, AR0102, Wuhan, China). Following SDS-PAGE separation, equal protein amounts were transferred to nitrocellulose membranes. After a 1-h block with 5% non-fat milk, membranes were treated with primary and secondary antibodies (LI-COR, 926–32,211, 1:20,000). The antibodies information were: anti-ZO-1 (Affinity, AF5145, 1:1000; Jiangsu, China), anti-Claudin-1 (Affinity, AF0127, 1:1000), anti-Occludin (Affinity, DF7504, 1:1000), anti-IL-6 (Affinity, DF6087 1:1000), anti-IL-1β (Affinity, AF5103, 1:1000), anti-IL-10 (Affinity, DF6894, 1:1000), anti-P65 (ABclonal, A19653, 1:5000; Wuhan, China), anti-P-P65 (ABclonal AP1294, 1:10,000), anti-β-actin (Bioss, bsm-33036 m, 1:10,000). Protein signals were captured and quantified via an infrared dual-color imaging system (LI-COR Biosciences, Lincoln, NE, USA). For each lane, a rectangular region of interest (ROI) of identical size was applied around the target band, and the integrated intensity was extracted following background subtraction. Signal intensities were normalized to β-actin for each lane to control for loading variability. All six biological replicates per group were included in the quantification and statistical analysis. For visual clarity in the figures, representative blots from three replicates per group are shown; the reported quantitative results, however, are based on data from all replicates.

### 2.8 16S rRNA gene sequencing and analysis

The microbial diversity in the jejunum was assessed via 16S rRNA gene sequencing. Total microbial DNA was isolated from jejunal content samples utilizing the QIAamp Fast DNA Stool Mini Kit (Qiagen, Hilden, Germany) following the protocol. DNA integrity and concentration were assessed via electrophoresis on a 1.0% agarose gel and spectrophotometry (NanoDrop2000, Thermo Scientific). All DNA samples were placed at −80 °C prior to downstream analysis. A preliminary PCR was conducted using randomly selected jejunal content samples to ensure successful amplification with minimal cycle numbers and appropriate product concentration. Once pretests were validated, a formal PCR was performed using Pro Taq polymerase and the V3–V4 region was amplified using primers 338F (5′-ACTCCTACGGGAGGCAGCAG-3′) and 806R (5′-GGACTACHVGGGTWTCTAAT-3′) (Guo et al., 2022). Each 20 μL reaction contained 2 × Pro Taq buffer, primers (0.4 μM final concentration each), template DNA (10 ng), and nuclease-free water. Purified amplicons were pooled in equal proportions and sequencing adapters were ligated to construct sequencing libraries. Sequencing was carried out using the Illumina HiSeq PE250 platform (Illumina, San Diego, USA) targeting the V3-V4 region. Following demultiplexing, raw paired-end reads were processed for quality control, filtering, and merging. The optimized sequences were denoised using the DADA2 algorithm (Callahan et al., 2016), yielding amplicon sequence variants (ASVs) with representative sequences and abundance tables.

### 2.9 Metagenomic analysis

Genomic DNA was isolated from the jejunal content using the Mag-Bind® Soil DNA Kit (Omega Bio-tek, USA), a protocol suited for high-complexity samples. DNA integrity and purity were assessed using 1% agarose gel electrophoresis. Fragmentation was performed using a Covaris M220 sonicator (Gene Company Limited, China) to yield ~350 bp fragments. Paired-end libraries were constructed, following standard procedures including adapter ligation, self-ligated fragments were removed via magnetic bead-mediated size selection, PCR amplification for template enrichment, and bead purification to obtain the final libraries. Sequencing libraries were run on a NovaSeq 6,000 system at Shanghai Majorbio Bio-Pharm Technology Co. using bridge-PCR cluster generation and sequencing-by-synthesis chemistry. Raw reads were filtered with fastp v0.20.0: reads < 50 bp, average Q < 20, or containing ambiguous bases were discarded and adapters trimmed (Chen et al., 2018). Host-derived sequences were then filtered out by aligning the clean reads against the sheep reference genome^1^using BWA v0.7.17 (Li and Durbin, 2009); reads showing high similarity to the host genome were discarded. Using MEGAHIT v1.1.2, high-quality reads were assembled *de novo*; contigs ≥ 300 bp were retained (Li et al., 2015). Coding sequences were predicted with Prodigal, and those ≥ 100 bp translated into proteins (Hyatt et al., 2010). Protein sequences were clustered with CD-HIT (90% identity and coverage) to generate a non-redundant gene catalogue, keeping the longest member of each cluster as representative (Fu et al., 2012). Gene representation profiles were generated by mapping clean reads to the non-redundant gene catalogue using SOAPaligner v2.21 at ≥ 95% sequence identity (Li et al., 2008). Taxonomic annotation employed DIAMOND searches against the NCBI NR database (E-value ≤ 1 × 10^−100^), followed by the lowest-common-ancestor algorithm to derive taxonomic profiles. Genes from bacterial taxa showing significant differential features among groups (LEfSe, LDA > 3, *p* < 0.05) were extracted and functionally annotated to infer microbial functional potential against the KEGG database^2^ using DIAMOND, with an E-value threshold of ≤ 1 × 10^−100^. KEGG pathway enrichment analysis was subsequently performed using the Majorbio Cloud Platform (Shanghai, China), with pathways identified as significantly altered based on a reporter score threshold of | ≥ 1.65| (Hong et al., 2024).

### 2.10 Bioinformatics analysis

All sequencing and bioinformatics analyses in this study were conducted by Shanghai Majorbio Bio-Pharm Technology Co., Ltd. (Shanghai, China). Alpha diversity indices were computed via the Mothur platform to evaluate microbial diversity within samples (Schloss et al., 2009). To assess differences in microbial community structure among groups, Principal Coordinates Analysis (PCoA) was carried out using Bray-Curtis distance metrics (Lozupone et al., 2011). Group-level variation in community composition was statistically assessed using Permutational Multivariate Analysis of Variance (PERMANOVA) (Anderson, 2001). To identify taxonomic biomarkers that differed among treatment groups, the Linear Discriminant Analysis Effect Size (LEfSe) method was applied with a threshold of LDA > 2 and *p* < 0.05 (Segata et al., 2011). Spearman analysis was further conducted to examine the associations between representative microbial features and the measured indicators, and the results were visualized using heatmap software tools.

### 2.11 Statistical analysis

Data were expressed as means ± standard error (SEM), and intergroup differences were determined using a one-way analysis of variance (ANOVA), followed by Tukey’s *post hoc* test for multiple comparisons, with significance assessed using GraphPad Prism version 10.0. Differences were considered significant at *p* < 0.05 and extremely significant when *p* < 0.01.

## 3 Results

### 3.1 Maternal dietary taurine supplementation increased offspring’s body weight

**Figure 1:**
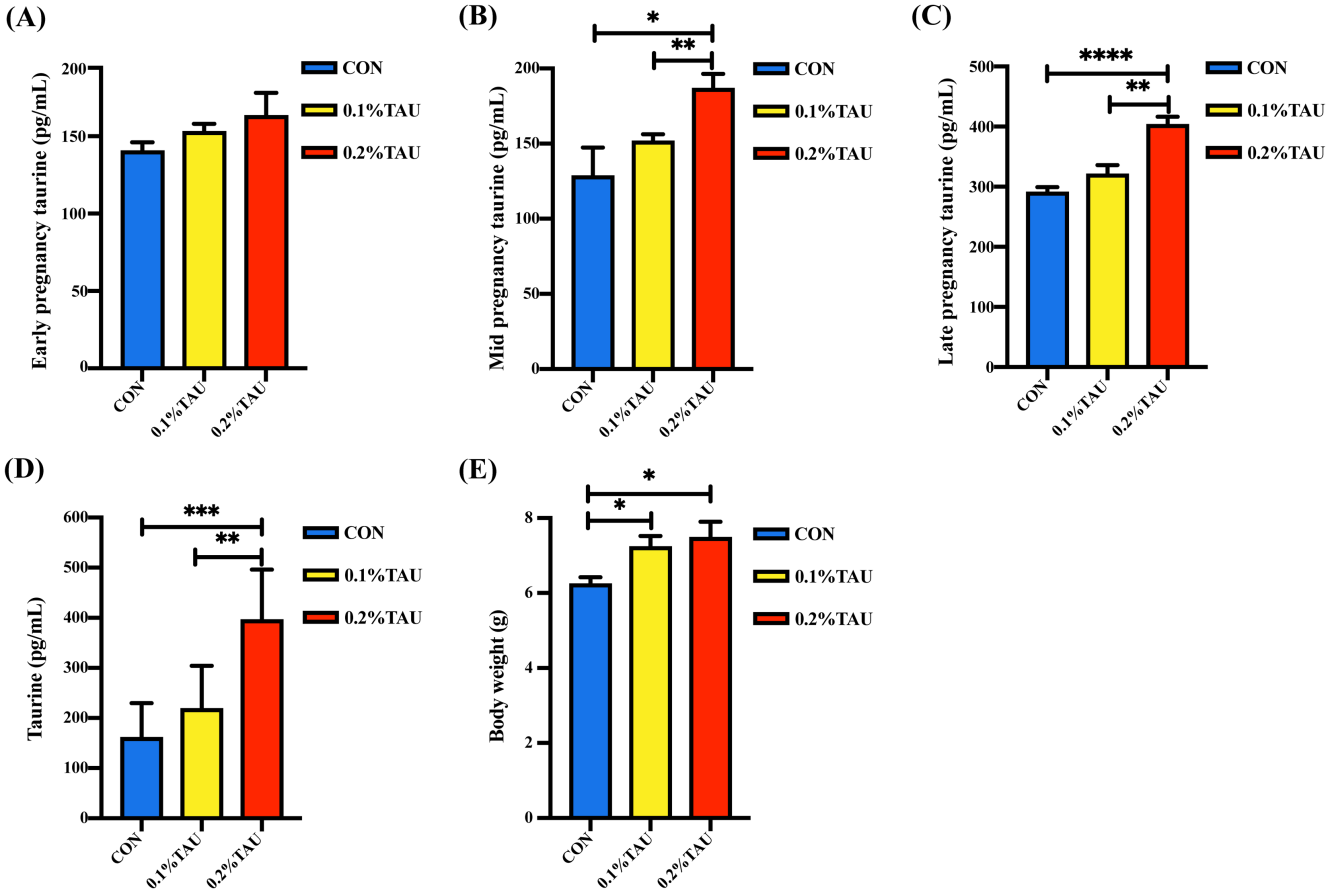
Maternal taurine supplementation during pregnancy promotes offspring development. Ewes were divided into three groups according to the feeding regimen: control group (CON), low-dose taurine group (0.1% TAU), and high-dose taurine group (0.2% TAU). **(A)** Taurine concentration in maternal serum during early pregnancy; **(B)** Taurine concentration in maternal serum during mid pregnancy; **(C)** Taurine concentration in maternal serum during late pregnancy; **(D)** Taurine concentration in offspring serum at 15 days of age; **(E)** Body weight of offspring. All data are presented as mean ± SEM. Statistical analysis was performed using one-way ANOVA followed by Tukey’s multiple comparison test (*p < 0.05, **p < 0.01, ***p < 0.001, ****p < 0.0001; *n* = 6 per group).

Although serum taurine levels in ewes were unchanged during early gestation, dietary taurine supplementation increased serum taurine concentrations in both middle and late gestation (Figures 1A–C). Similarly, the bile acid levels were increased in taurine fed ewes and their offspring lambs (Supplementary Figures S1A–D). Moreover, the serum taurine abundances in offspring at 15 days of age were elevated in a dose-dependent manner (Figure 1D). Notably, compared with the control group, the offspring’s BW in the taurine-fed groups was elevated (Figure 1E).

### 3.2 Intestinal morphology and barrier integrity in lambs were changed by maternal taurine supplementation

**Figure 2:**
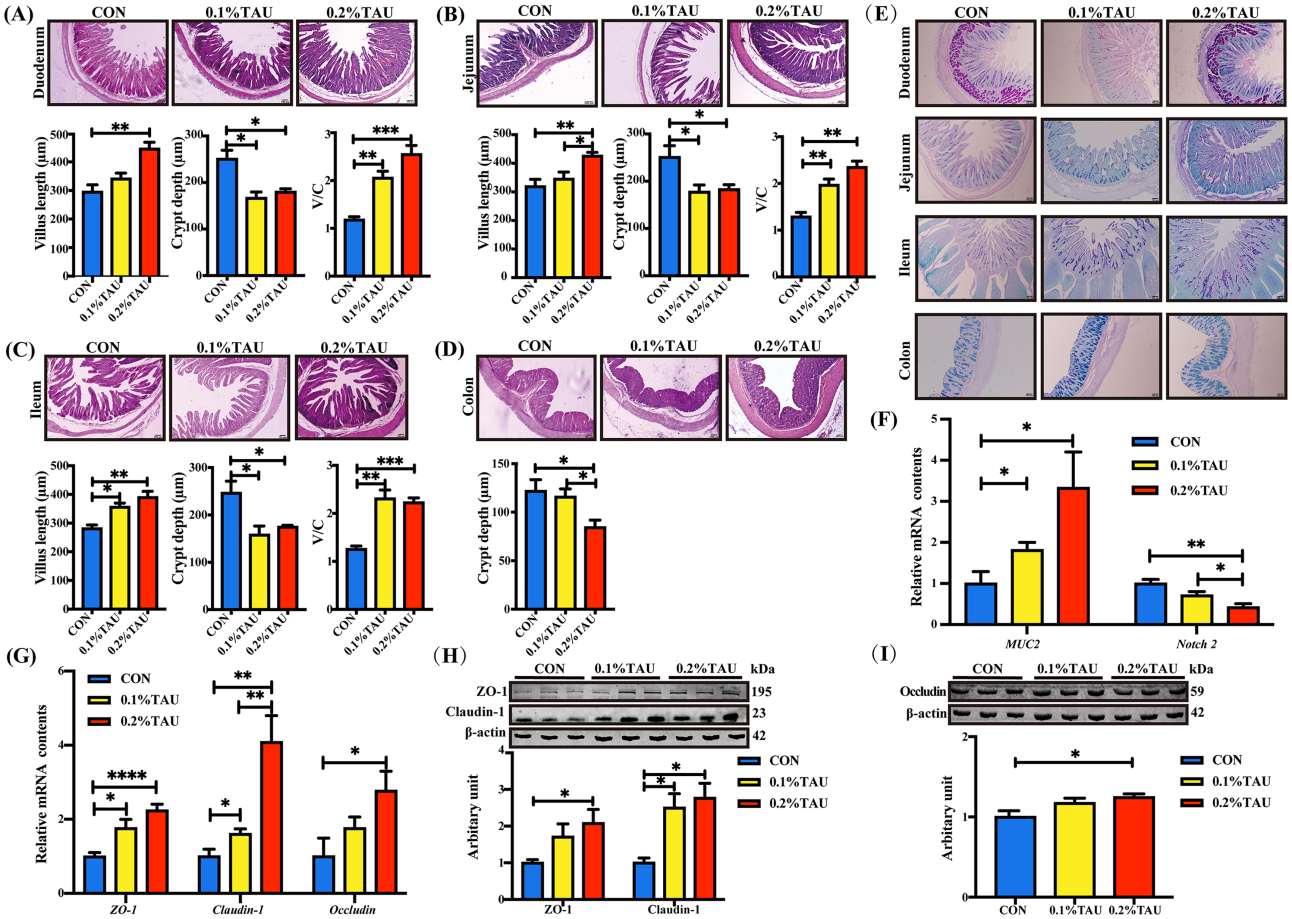
Maternal dietary taurine supplementation during gestation promotes the maturation of intestinal structure and barrier function in offspring lambs. **(A–D)** H&E staining of the duodenum, jejunum, ileum, and colon, along with measurements of villus height, crypt depth, and villus-to-crypt ratio; **(E)** AB-PAS staining for goblet cells and mucin secretion in intestinal tissues; **(F)** Relative mRNA expression of *MUS2* and *Notch2*; **(G–I)** Expression of tight junction proteins (ZO-1, Claudin-1, and Occludin) at the protein and mRNA levels. Western blot images show three representative lanes per group for visualization; all six biological replicates were quantified and included in the statistical analyses. All data are presented as mean ± SEM. Statistical analysis was performed using one-way ANOVA followed by Tukey’s multiple comparison test (*p < 0.05, **p < 0.01, ***p < 0.001, ****p < 0.0001; *n* = 6 per group for all analyses).

Lambs in taurine-treated groups displayed increased villus length and villus-to-crypt ratio, along with decreased crypt depth compared to the control group in a dose-dependent manner (Figures 2A–D). AB-PAS staining results suggested an increased number of goblet cells in the intestinal epithelium of offspring from those taurine-fed ewes (Figure 2E). As expected, the *MUC2* mRNA expression was markedly elevated in treatment lambs (Figure 2F). Furthermore, the abundances of tight junction proteins and mucin-related factors, including ZO-1, Claudin-1, and Occludin in treatment groups were elevated at the mRNA level, with consistent trends observed at the protein level (Figures 2G–I). In addition, the expression of *Notch2* was decreased by maternal dietary taurine supplementation.

### 3.3 Immune responses and antioxidant capacity were enhanced in intestine by maternal taurine supplementation

**Figure 3:**
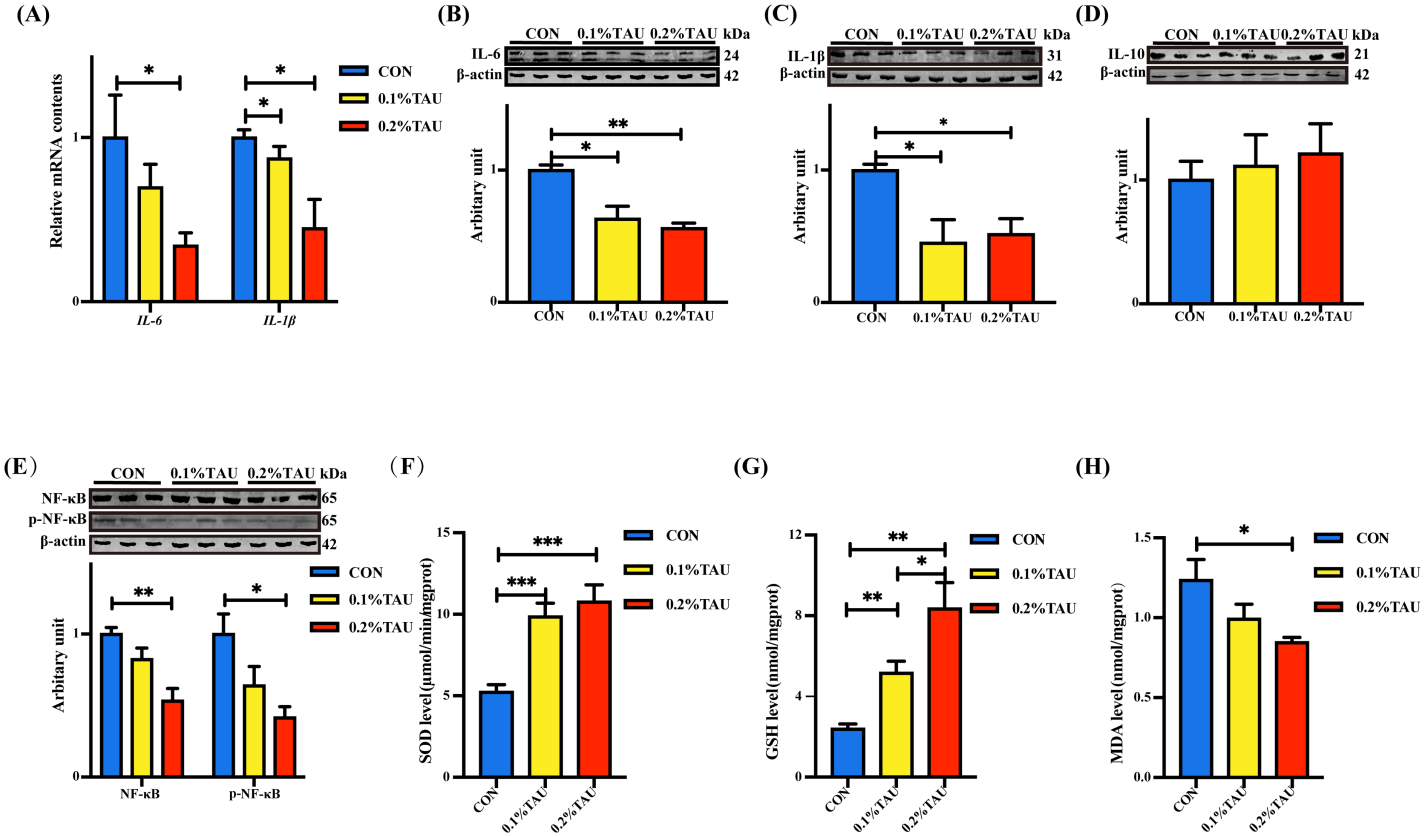
Maternal taurine supplementation during pregnancy enhanced antioxidant capacity and modulated intestinal immune responses in the offspring lambs, contributing to improved gut health. **(A)** Relative mRNA expression levels of *IL-6* and *IL-1β*; **(B–E)** relative protein expression levels of IL-6, IL-1β, IL-10, and NF-κB/phospho-NF-κB; western blot images show three representative lanes per group for visualization; all six biological replicates were quantified and included in the statistical analyses. **(F–H)** Superoxide dismutase (SOD) activity, glutathione (GSH) levels, and malondialdehyde (MDA) concentration. All data are presented as mean ± SEM. Statistical analysis was performed using one-way ANOVA followed by Tukey’s multiple comparison test (*p < 0.05, **p < 0.01, ***p < 0.001; *n* = 6 per group for all analyses).

The mRNA abundance of *IL-6* and *IL-1β*, were reduced in intestines of taurine-treated group compared to the control lambs (Figure 3A), and these trends were supported by the Western blot results (Figures 3B,C). Protein abundance of IL-10 was increased in treatment groups, but the difference was not statistically significant (Figure 3D). Meanwhile, both NF-κB and p-NF-κB contents were decreased in intestines of lambs from taurine-supplemented ewes (Figure 3E). Moreover, the SOD and GSH contents in intestine were elevated, while the MDA levels were decreased in treatment group (Figures 3F–H).

### 3.4 Modulation of gut microbial composition in offspring by maternal taurine supplementation

To evaluate the effects of maternal taurine intervention on the gut microbial composition of offspring, 16S rRNA gene sequencing was performed. After quality control and denoising with DADA2, ASV tables were generated and used for downstream analyses.

**Figure 4:**
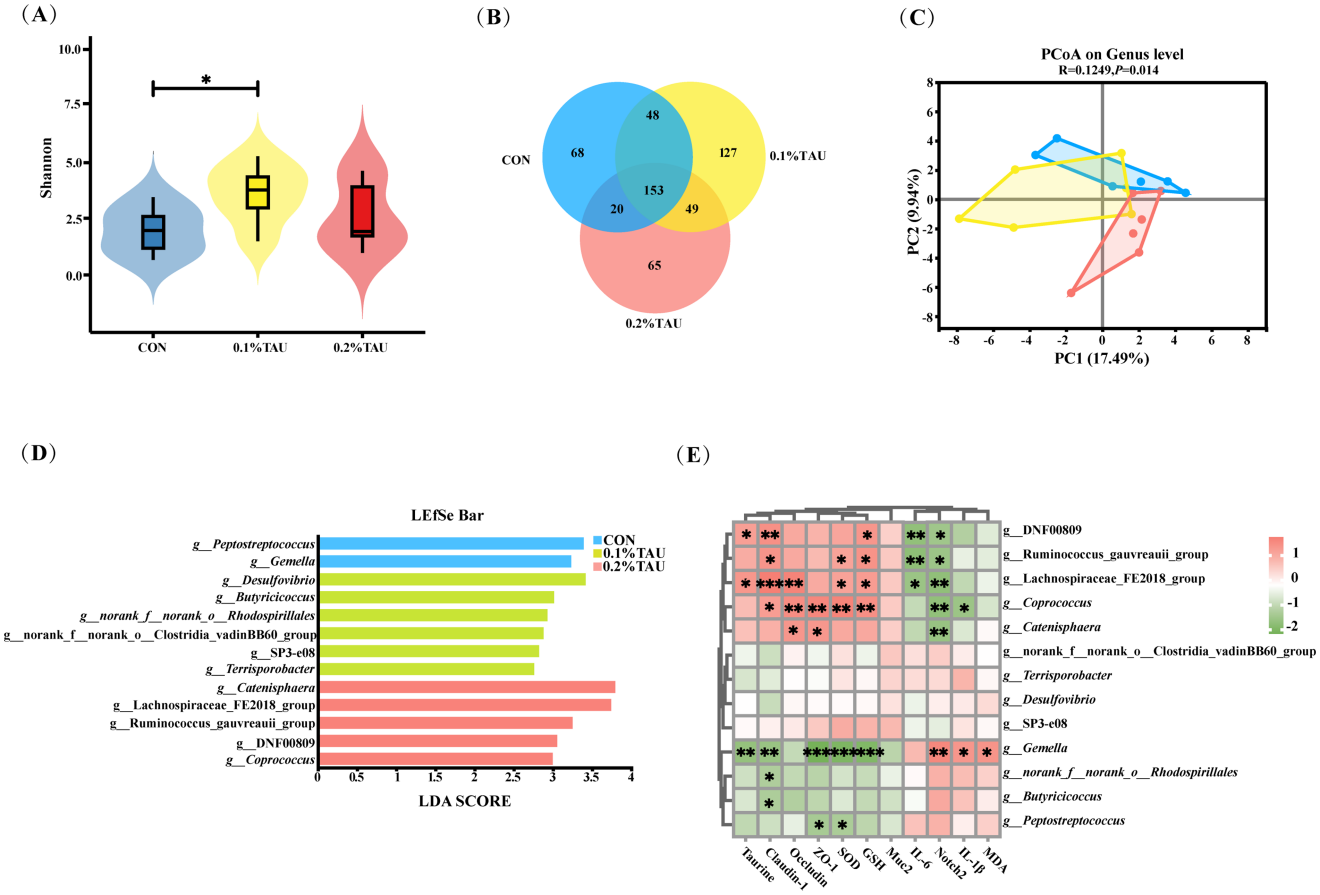
Maternal taurine supplementation during pregnancy optimized the gut microbiota composition in offspring lambs. **(A)** Shannon index indicating microbial diversity among groups. Statistical analysis was performed using one-way ANOVA followed by Tukey’s multiple comparison test; **(B)** Venn diagram showing the number of shared and unique ASVs among groups; **(C)** Principal coordinates analysis (PCoA) illustrating the overall microbial community structure among groups; **(D)** LEfSe analysis identifying significantly different taxa among groups (LDA > 2, *p* < 0.05); **(E)** Spearman correlation analysis between LEfSe-identified genera and intestinal health-related parameters (*p < 0.05, **p < 0.01, ***p < 0.001; *n* = 6 per group).

A total of 530 representative ASVs were detected across all samples, with 289, 377, and 287 ASVs detected in the CON, 0.1% TAU, and 0.2% TAU groups, respectively. Among these, 153 core ASVs were shared across all three groups. These shared ASVs, which were assigned to 153 genera, were used to generate the Venn diagram at the genus level (Figure 4B). The majority of sequences in each group were accounted for by these shared ASVs, indicating a relatively stable core microbiota, while taurine-related effects were primarily reflected in taxon-specific differences rather than in the number of shared ASVs. PCoA analysis at the genus level did not show a clear separation among the three groups, with PC1 and PC2 explaining 17.49 and 9.94% of the total variation (Figure 4C), indicating that maternal taurine supplementation did not induce a pronounced shift in overall microbial community structure. The *α*-diversity analysis showed that the Shannon index was significantly increased in the 0.1% TAU group compared with the control group (*n* = 6 per group) (Figure 4A). No statistically significant difference was observed in the 0.2% TAU group. Other α-diversity indices were also calculated but did not show statistically significant differences (Supplementary Figures S2A–D).

LEfSe analysis was performed to identify discriminative bacterial taxa among the three groups based on both statistical significance and effect size (Figure 4D) (LDA > 2, *p* < 0.05). A total of 13 genera were identified as group-specific biomarkers. The control group was characterized by *Peptostreptococcus* and *Gemella*, genera that have been associated with intestinal inflammation or pathogenic potential. In the 0.1% TAU group, several genera, including *Desulfovibrio*, *Butyricicoccus*, and *Terrisporobacter,* were identified as discriminative features. Notably, *Butyricicoccus* has been reported to produce butyrate and contribute to intestinal barrier function (Xiao et al., 2023; Kwon et al., 2024). The 0.2% TAU group was characterized by *Coprococcus*, Ruminococcus_gauvreauii_group, and Lachnospiraceae_FE2018_group, taxa previously associated with antioxidant capacity, anti-inflammatory responses, and intestinal homeostasis.

Spearman correlation analysis was performed between the LEfSe-identified differential genera and the aforementioned phenotypic indices. Several genera enriched in the treatment groups, particularly those in the 0.2% TAU group, exhibited significant correlations with gut health-related indicators (Figure 4E). Although correlations in the 0.1% TAU group did not reach statistical significance, several phenotypic indicators already showed clear improvement, suggesting a trend toward beneficial associations (given the modest sample size, some correlations may not reach statistical significance despite observable trends, *n* = 6 per group). In contrast, genera enriched in the control group were generally positively associated with unfavorable phenotypes and negatively associated with intestinal barrier and antioxidant indices.

### 3.5 Metagenomic analysis of changes in gut microbiota composition in offspring

**Figure 5:**
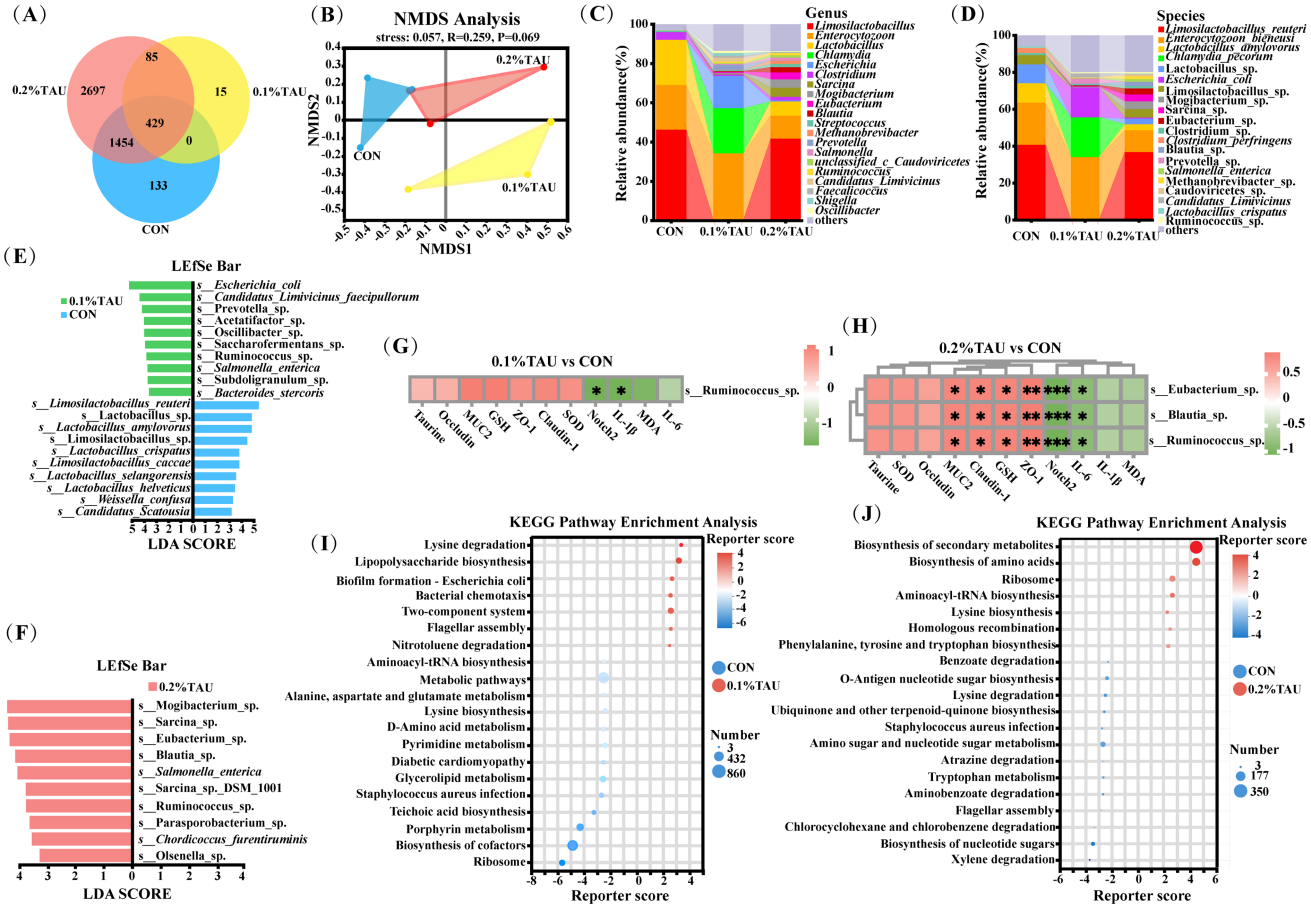
Metagenomic analysis indicated associations between maternal taurine supplementation and differences in microbial features and functional potential in the offspring gut microbiota. **(A)** Venn diagram showing shared and group-specific microbial features among groups; **(B)** non-metric multidimensional scaling (NMDS) based on Bray-Curtis distances illustrating overall differences in microbial feature profiles; **(C)** distribution of dominant genera; **(D)** distribution of dominant species; **(E,F)** LEfSe analysis identifying discriminative taxa among groups (LDA > 3, *p* < 0.05); **(G,H)** spearman correlation analysis between representative bacterial genera and phenotypic parameters (*p < 0.05, **p < 0.01, ***p < 0.001; significance refers only to these correlation analyses); **(I,J)** differences in microbial functional potential inferred from KEGG pathway analysis in the jejunal microbiota of neonatal lambs in the 0.1 and 0.2% taurine groups compared with the control group. Gene annotations were conducted using DIAMOND against the KEGG database with an E-value threshold of ≤ 1 × 10^−100^, and significantly altered pathways were defined by a reporter score of | ≥ 1.65| (etagenomic samples, *n* = 3 per group).

Venn diagram analysis identified a total of 429 core microbial features shared among the three groups (Figure 5A). The number of group-specific microbial features observed in the 0.2% TAU group was higher than that in the control group. While this difference may be partially influenced by technical factors such as sequencing depth or assembly sensitivity, the consistent presence of these group-specific features across replicates indicates they may be of interest for further study alongside dominant taxonomic features. To assess overall differences in microbial community structure among the groups, non-metric multidimensional scaling (NMDS) was performed to visualize *β*-diversity. NMDS analysis suggested modest differences in microbial community structure among the three groups (Figure 5B).

At the genus level, *Limosilactobacillus*, *Enterocytozoon*, *Lactobacillus*, *Chlamydia*, *Escherichia*, *Clostridium*, *Sarcina*, *Mogibacterium*, *Eubacterium*, *Blautia* were the dominant genera (Figure 5C). Similar distribution patterns were observed at the species level (Figure 5D). Several genera, including *Eubacterium*, *Blautia*, *Ruminococcus*, and *Prevotella* showed higher representation in the treatment groups. LEfSe analysis further identified bacterial species discriminating among groups (Figures 5E,F) (LDA > 3, *p* < 0.05), several of which have been previously associated with gut health in prior studies. It is worth noting that, alongside Ruminococcus_sp., the 0.1% TAU group also harbored detectable levels of certain taxa, including *Escherichia coli* and *Salmonella enterica*, which are occasionally associated with opportunistic infections under specific conditions, potentially indicating an altered gut microbial configuration at this dosage. In contrast, the 0.2% TAU group maintained a more stable profile of treatment-responsive beneficial taxa.

Using Spearman correlation analysis, we focused on Eubacterium_sp., Ruminococcus_sp. and Blautia_sp., taxa that showed higher representation in the treatment groups, and examined their relationships with the aforementioned phenotypic indicators. In the 0.2% TAU group, significant positive correlations were observed with intestinal barrier and antioxidant-related indicators such as MUC2, Claudin-1, GSH, and ZO-1, and significant negative correlations with inflammation-related markers such as Notch2 and IL-6 (Figures 5G,H). In the 0.1% TAU group, the overall correlation patterns were directionally consistent with those in the 0.2% TAU group. Notch2 and IL-1β also showed negative associations with Ruminococcus sp., and other phenotypic indicators followed similar trends, although none reached statistical significance. At the functional level, KEGG pathway analysis revealed distinct microbial metabolic profiles among the three groups (Figures 5I,J). The top 20 differentially represented pathways ranked by reporter score are shown in the figure. In the 0.1% TAU group, pathways with positive reporter scores were primarily associated with microbial adaptability and core metabolic processes, including two-component system, lysine degradation, nitrotoluene degradation, lipopolysaccharide biosynthesis, biofilm formation by *Escherichia coli*, bacterial chemotaxis, and flagellar assembly. In contrast, the 0.2% TAU group exhibited a broader set of biosynthetic pathways with positive reporter scores relative to the control group, such as the biosynthesis of amino acids, aminoacyl-tRNA biosynthesis, lysine biosynthesis, and phenylalanine, tyrosine, and tryptophan biosynthesis. These results suggest that moderate taurine supplementation was associated with distinct shifts in microbial functional potential related to adaptability and metabolic capacity.

## 4 Discussion

Diarrhea in neonatal lambs impairs intestinal function, nutrient absorption, growth potential, ultimately reducing survival rates and rearing efficiency (Shi et al., 2022). Since diarrhea often develops from intestinal dysfunction, early interventions targeting the gut may offer preventive benefits. In this context, regulating neonatal gut health through maternal nutrition during gestation represents a promising strategy. This study addresses the issue of neonatal diarrhea in lambs by examining the effects of maternal taurine supplementation on newborns, with specific evaluations of body weight, intestinal microbiota, intestinal morphology, and barrier function. Amino acids supplementation have received increasing attention for their roles in supporting intestinal integrity and microbiota stability (Beaumont and Blachier, 2020). In addition, metabolites derived from the microbial metabolism of tryptophan, amino acids with branched-chain structures and those containing sulfur groups have been reported to promote intestinal health (Venkatesh et al., 2014; Yang et al., 2016).

In the liver, taurine conjugates with bile acids, enhancing their emulsification capacity and promoting the breakdown and absorption of fat-soluble compounds (Duszka, 2022; Miyazaki et al., 2023). We found that lambs born to taurine-supplemented ewes during gestation exhibited greater BW at 15 days of age. Considering that BW advantages gained during the first three weeks are often sustained throughout the fattening phase, resulting in greater average daily gains and improved carcass characteristics (Zhang et al., 2023; Akyüz et al., 2025), these findings suggest that maternal taurine supplementation may enhance the growth potential of offspring lambs. Given the critical role of intact intestinal morphology in supporting growth performance, we hypothesized that intestinal integrity and/or function were imporved in lambs. As expected, an increase in villus length and a decrease in crypt depth were observed. Such morphological improvements expanded the effective surface area of the intestinal lining and simultaneously enhanced the activity of brush-border enzymes, thereby improving the absorption efficiency of nutrients such as glucose and amino acids (Zhou R. et al., 2024). Indeed, villus height has been shown to correlate with average daily gain (Li X. et al., 2025). Conversely, villus atrophy and impaired nutrient transport have been closely linked to postnatal growth retardation (Jiang et al., 2025). Further analysis suggested that the improvement in villus morphology might be associated with enhanced gut mucosal barrier function, as shown by increased goblet cell numbers and upregulated expression of Tight Junction (TJ) proteins, such as Occludin, Claudin-1, and ZO-1. The reinforcement of barrier function establishes a more stable intestinal microenvironment and provides structural support for well-organized villus development, ultimately contributing to optimal physiological status and sustained growth performance (Zhao et al., 2023). Thus, maternal taurine intake may contribute to early-life weight advantages in lambs, which might be attribueted to the improved villus architecture and enhanced nutrient absorption efficiency.

Intestinal inflammation has been shown to impair the expression and localization of TJ proteins, including Occludin and ZO-1, thereby compromising intestinal barrier integrity (Al-Sadi et al., 2008; Capaldo and Nusrat, 2009). For instance, inflammatory stimuli in weaned piglets increase intestinal permeability, while nutritional interventions that mitigate inflammation can help restore epithelial structure and barrier integrity (Wen et al., 2020). Therefore, the enhancement of the intestinal mucosal barrier observed in this study may also be associated with improvements in immune status and oxidative stress. We found that maternal taurine supplementation reduced the expression of IL-6 and IL-1β in the jejunal tissue of lambs, along with suppression of NF-κB signaling, while upregulating the level of IL-10. These findings indicate that modulation of the local immune microenvironment may contribute to the stabilization and regeneration of the gut barrier.

Oxidative stress is a critical contributor to the impairment of intestinal barrier function, as excessive ROS can damage epithelial cells structure and induce apoptosis. In contrast, enhanced antioxidant defenses help preserve the integrity of intestinal mucosal structure and function (Li B. et al., 2025). Taurine has been reported to mitigate oxidative stress-induced damage: Sandoghdar et al. (2024) observed that taurine added to the diet reduced oxidative stress in early-aged broiler chickens and Zhou M. et al. (2024) demonstrated that taurine improved antioxidant enzyme activity and reduced oxidative damage in stressed weaned piglets. Consistent with these findings, our study showed that maternal taurine intake significantly improved the intestinal antioxidant profile of neonatal lambs. The simultaneous reduction in local inflammatory responses and enhancement of oxidative defense may work in concert to strengthen the mucosal barrier and stabilize the epithelial microenvironment.

Although maternal taurine supplementation did not induce a pronounced shift in overall microbial community structure, it was associated with the identification of discriminative bacterial taxa in neonatal lambs based on LEfSe analysis. These discriminative taxa mainly included particularly members of *Lachnospiraceae*, *Ruminococcus*, and *Eubacterium*, which have been reported to contribute to intestinal homeostasis. Functional annotation based on KEGG pathway analysis suggested differences in microbial functional potential across taurine supplementation levels. At 0.1% taurine supplementation, microbial functions were modestly shifted toward pathways involved in microbial adaptability and core metabolic processes, including two-component system and lysine degradation, etc. These functional shifts likely support microbial adjustment to the jejunal environment and core metabolic functions. In contrast, the 0.2% taurine group was characterized by broader representation of biosynthetic pathways, including amino acid, aminoacyl-tRNA, lysine, and aromatic amino acid biosynthesis, reflecting a distinct microbial functional potential related to biosynthesis, which has been reported to be associated with intestinal epithelial maintenance and redox balance. Although typically dominant in the colon, *Lachnospiraceae* and *Ruminococcaceae* can be transiently detected in the jejunum during early life and possess the genomic capacity to ferment glycans into SCFAs and other metabolites that, based on prior studies, may contribute to mucosal immunity, redox homeostasis, and epithelial integrity (Rubio-Del-Campo et al., 2021; He and Dong, 2023; Huang et al., 2023). These microbial metabolites fuel epithelial energy needs, stimulate tight junction protein expression, and help preserve intestinal structural integrity (Yan and Ajuwon, 2017; Matheus et al., 2023). Moreover, these commensals have been reported to suppress NF-κB-mediated inflammatory responses while activating the Nrf2 antioxidant signaling pathway, which may be associated with reduced pro-inflammatory responses and enhanced activities of antioxidant enzymes (Ali et al., 2022; Chen et al., 2024). Certain members of the *Eubacterium* have also been reported to modulate mucosal immunity and scavenge reactive oxygen species (Lu et al., 2022; Bai et al., 2024). Additionally, alterations in *Blautia* and *Coprococcus* abundance have been positively associated with increased levels of anti-inflammatory cytokines (Liu et al., 2021; Yang et al., 2023). Although the underlying mechanisms remain to be fully elucidated, taurine has been shown to shape gut microbial composition by improving the mucosal metabolic environment, regulating redox status, or acting as a precursor in bile acid synthesis (Duszka, 2022; Weike et al., 2023). Collectively, these taurine-associated microbiota changes align with the observed reductions in intestinal inflammation and oxidative stress, and may contribute to the improved mucosal barrier function.

Although the present study identified associations between maternal taurine supplementation and alterations in the jejunal microbiota of neonatal lambs, the sample size used is typical for controlled livestock nutrition trials and remains limited for capturing the full complexity of microbiome-associated variation. Future studies with larger cohorts and longitudinal sampling are warranted to validate these findings and further elucidate the functional roles of specific microbial taxa.

## 5 Conclusion

Maternal dietary taurine supplementation during pregnancy was associated with improved indicators of early-life gut health in neonatal lambs, alongside increased representation of gut microbial taxa frequently associated with gut health in ruminants, including members of *Lachnospiraceae*, *Ruminococcus*, and *Eubacterium*. Concurrently, differences in microbial functional potential, changes in intestinal immune homeostasis and antioxidant status, and enhanced indicators of intestinal barrier maturation were observed. Together, these findings underscore the potential of maternal nutritional programming as a strategy to support intestinal development and gut-related physiological stability during early life, with possible implications for pre-weaning health outcomes.

## Data Availability

The datasets presented in this study can be found in online repositories. The metagenomic data are available in the NCBI Sequence Read Archive under accession number: PRJNA1280053, and the 16S rRNA sequencing data are available under accession number: PRJNA1277452.

## Footnotes

1
https://www.ncbi.nlm.nih.gov/datasets/genome/?taxon=9940

2
http://www.genome.jp/kegg/
