# Supplementary material for: Maternal dietary taurine supplementation improves intestinal health of lambs via modulating gut microbiota and barrier function
Source: Front Microbiol. 2026 Feb 16;17:1662296. doi: 10.3389/fmicb.2026.1662296 (PMC12950800; doi:10.3389/fmicb.2026.1662296)
Supplement: Supplementary file 1 [file Supplementary_file_1.docx]

Supplementary Material

**Supplementary figures**

**Fig. S1**

**
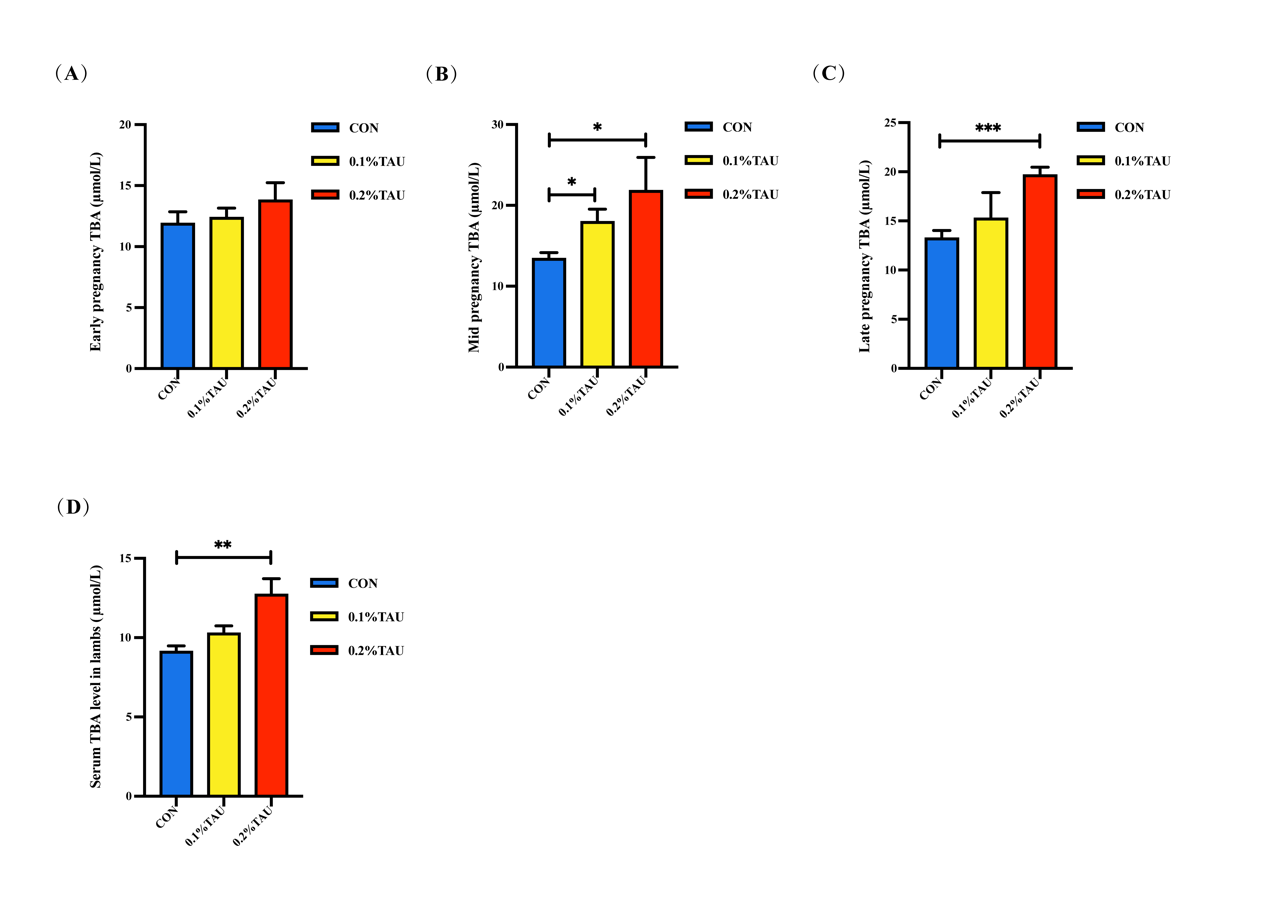
**

**Supplementary Figure 1.** Changes in bile acid levels during different gestational stages. (A) Bile acid concentration in maternal serum during early pregnancy; (B) Bile acid concentration in maternal serum during mid pregnancy; (C) Bile acid concentration in maternal serum during late pregnancy; (D) Bile acid concentration in offspring serum at 15 days of age. All data are presented as mean ± SEM. Statistical analysis was performed using one-way ANOVA followed by Tukey’s multiple comparison test. (**P*<0.05, ***P*<0.01, ****P*<0.001; n = 6).
