# Supplementary material for: Maternal dietary taurine supplementation improves intestinal health of lambs via modulating gut microbiota and barrier function
Source: Front Microbiol. 2026 Feb 16;17:1662296. doi: 10.3389/fmicb.2026.1662296 (PMC12950800; doi:10.3389/fmicb.2026.1662296)
Supplement: Supplementary file 2 [file Supplementary_file_2.docx]

Supplementary Material

**Supplementary figures**

**Fig. S2**


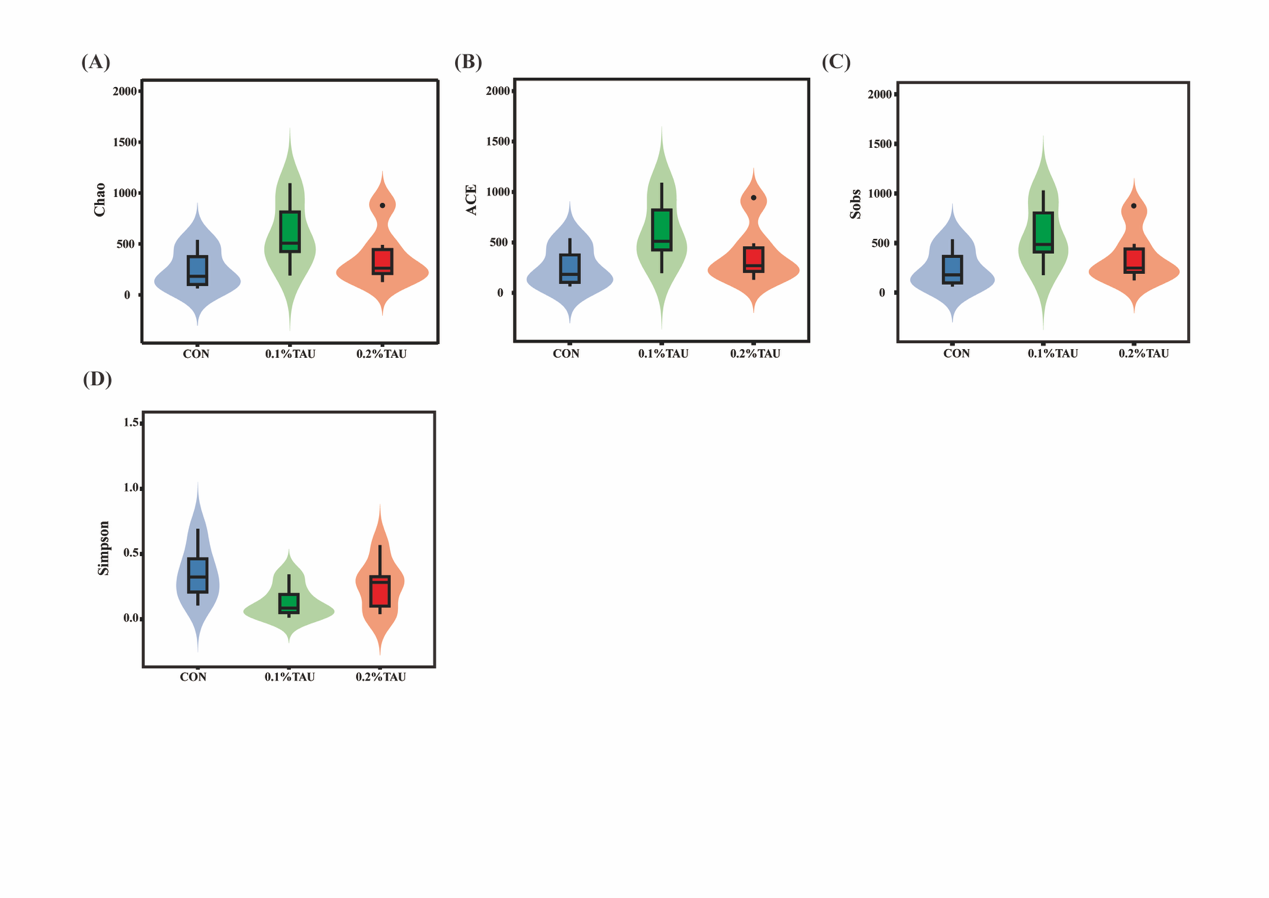


**Supplementary Figure 2.** α-diversity indices of the jejunal microbiota other than the Shannon index. (A) Chao index; (B) ACE index; (C) Sobs index; (D) Simpson index. (n = 6 per group).
